# Advanced Surface Color Quality Assessment in Paper-Based Full-Color 3D Printing

**DOI:** 10.3390/ma14040736

**Published:** 2021-02-04

**Authors:** Jieni Tian, Jiangping Yuan, Hua Li, Danyang Yao, Guangxue Chen

**Affiliations:** 1 State Key Laboratory of Pulp and Paper Engineering, South China University of Technology, Guangzhou 510640, China; Tianjieni123@126.com (J.T.); li20160808h@163.com (H.L.); dyyao9722@163.com (D.Y.); 2 Institute for Visualization and Data Analysis, Karlsruhe Institute of Technology, 76131 Karlsruhe, Germany

**Keywords:** 3D printing, surface property, quality evaluation, color printing, image quality metric

## Abstract

Color 3D printing allows for 3D-printed parts to represent 3D objects more realistically, but its surface color quality evaluation lacks comprehensive objective verification considering printing materials. In this study, a unique test model was designed and printed using eco-friendly and vivid paper-based full-color 3D printing as an example. By measuring the chromaticity, roughness, glossiness, and whiteness properties of 3D-printed surfaces and by acquiring images of their main viewing surfaces, this work skillfully explores the correlation between the color representation of a paper-based 3D-printed coloring layer and its attached underneath blank layer. Quantitative analysis was performed using ΔE*_ab_, feature similarity index measure of color image (FSIMc), and improved color-image-difference (iCID) values. The experimental results show that a color difference on color-printed surfaces exhibits a high linear correlation trend with its FSIMc metric and iCID metric. The qualitative analysis of microscopic imaging and the quantitative analysis of the above three surface properties corroborate the prediction of the linear correlation between color difference and image-based metrics. This study can provide inspiration for the development of computational coloring materials for additive manufacturing.

## 1. Introduction

Three-dimensional printing is an innovative technology that is widely used for personalized digital fabrication [1]. With great advances in new processes and materials, the manufacturing of functional 3D-printed parts is becoming more and more widespread. However, no corresponding progress has been made in color 3D printing and 3D printing color reproduction, making the surface color management of 3D-printed parts still a technical bottleneck for the industry [2]. This is mainly due to the lack of accurate color reproduction theory and evaluation system for color 3D printing, for which the 3D printing academic community has conducted several academic conferences to discuss the standardization of color 3D printing process [3].

Subsequently, the concept of full-color 3D printing was introduced and the corresponding paper-based, powder-based, and plastic-based full-color 3D printers were developed and released [4,5,6]. Full-color 3D printing not only refers to multi-color printing but also emphasizes more accurate color reproduction of color 3D prints [7]. It is an advanced stage in the development of color 3D printing. According to the standard definition of additive manufacturing principles, the following principles are more likely to achieve full-color 3D printing: binder jetting (BJ), sheet lamination (SL), material jetting (MJ), and vat photopolymerization (VPPM) [8]. 

Among them, paper-based full-color 3D printing based on the SL principle exhibits significant advantages including being eco-friendly, colorful, and low cost to expand its applications in the field of cultural creativity [9,10]. In recent years, paper-based 3D printing materials have been upgraded from single-sheet paper to roll paper with a further reduction in printing time. Meanwhile, the double-sided coloring of the paper substrate has been changed to single-sided coloring, resulting in higher criteria for the ink penetration behavior and surface properties of the paper substrate. Moreover, the massive applications of paper-based full-color 3D printing in the field of cultural and creative industries have also given rise to online evaluations of surface color reproduction, remaining a tricky challenge [11].

There are many factors that influence the quality of color reproduction throughout the full-color 3D printing process, but paper-based full-color 3D printing color reproduction is easier to approach than the color evaluation criteria of the 2D printing field [12,13,14,15]. However, the complex geometry and uneven optical characteristics of 3D-printed products and even more material components lead to the fundamental differences between full-color 3D printing and 2D printing [16]. For example, the color accuracy control of vertical surfaces (Z-axis) in color 3D model printing is a new challenge.

The evaluation of color reproduction of color 3D-printed parts is also a hot research topic in the field of 3D printing materials and color science. Xiao and Sun developed a color reproduction system for the precise and automated processing of silicone-based soft tissue prostheses based on powder-based 3D printing, evaluated the color reproducibility of this system by measuring ΔE^*^_ab_ and spectral similarity, and provided an outlook on the color accurate reproduction evaluation scheme of full-color 3D printing from the perspective of attached color image reproduction [17,18,19,20,21]. Fastowicz et al. presented image-based metrics including entropy, histogram equalization, Hough transform, structural similarity, and feature similarity to evaluate the surface quality classification of single-color 3D prints in high- and low-quality printing modes using an FDM (Fused Deposition Modeling) 3D printer [22,23,24,25]. Urban et al. utilized an MJ printer to revise the subjective scale model of the transparency properties of plastic-based full-color 3D printing on its color reproduction quality from the precise control of transparency in light-curing resin molding [26,27]. Although these interesting studies explore the control of color reproduction on different substrates, the objective evaluation metrics used differ and the associations between these metrics are not yet clear.

For this reason, our 3D printing team has also conducted some empirical explorations for this gap. Wang et al. analyzed the relationship between the microscopic image and color difference of powder-based full-color 3D prints and proposed a microscopic image analysis method to improve color reproduction [28]. Yuan et al. evaluated the influence of the thickness and coloring order of the plastic-based full-color 3D prints on the color difference and mean structural similarity (MSSIM) and discussed the association between the two metrics, but no significant linear trend was found [29]. So far, there is no unified standard for accurate and comprehensive evaluation or a system for the color reproduction of full-color 3D prints [30].

In order to enrich the study of the influence of substrates on the evaluation of color reproduction quality in full-color 3D printing, the Mcor ArkePro paper-based full-color 3D printer and specific test model design are tested for empirical analysis of objective evaluation. The photographing image quality is evaluated by the color and texture information of the standard acquisition of 3D-printed sample surface and quantitatively correlated with the corresponding coloring position. Then, the correlation of the roughness, glossiness, and whiteness of the printed surface on the corresponding color reproduction are explored in combination with the microscopic image analysis to understand the effect of objective metrics on the surface color efficiency evaluation of paper-based 3D printing.

## 2. Materials and Methodology

**Figure 1:**
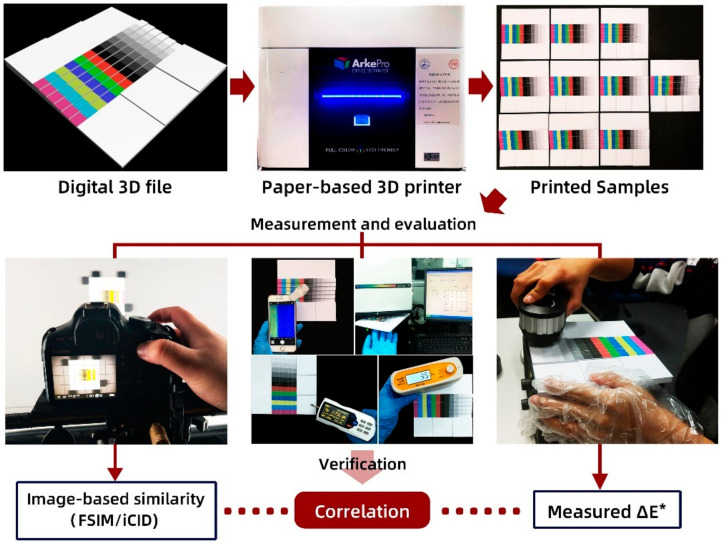
The flowchart of our experiment.

In this study, the flow chart of our experiment is illustrated in Figure 1. It consists of four stairs: the first is specific 3D color test mode design and printing; the second is the evaluation of measured surface attributes including chromaticity, roughness, glossiness, and whiteness; the third is the capture and computation of the image-based metrics; and the fourth is the correlation analysis between color difference and image-based metrics as well as microscopic features verification. 

The 3D modeling software used in this experiment is Autodesk 3ds Max (Autodesk, San Rafael, CA, USA). The 3D printer is the Mcor ArkePro paper-based full-color 3D printer (Mcor-technologies, Dublin, Ireland) with a layer accuracy of 0.1 mm. The color difference of sampling color was automatically executed by a portable eXact spectrophotometer (X-Rite, Grand Rapids, MI, USA). The HD (High-Definition) imaging acquisition was performed by a Canon EOS 500D (Canon Co., Ltd, Oita Prefecture, Kunisaki, Japan) with Canon 0.35 mm/1.1ft-∞ lens. The microscopic images were captured by a handheld version of the XY9100-100X microscope (GUXIANG Optical Co., Ltd, Shanghai, China). The whiteness measurements were performed by a Color-Touch PC CTP-ISO Whiteness Meter (Technidyne, New Albany, IN, USA) with a 2° field of view and D65 light source. The SFMIT MIT-60° and TR200 (SanFeng, Changzhou, Jiangsu, China) were used for the surface glossiness and roughness.

### 2.1. Model Design and Color Parameters

Ten 3D color test models are designed with 6 white stairs and 6 color stairs. As a blank control, the 6 white stairs were designed one slice layer lower than the corresponding 6 color stairs to ensure that the surface performance of the white block covered by the color bar was consistent. Since the test specimens were very thin, all the paper-based test models had an additional base to enhance their mechanical strength. The thickness of each color stair and white block on the paper-based test model with specific bases is shown in Table 1. A white stair corresponds to a white block.

Each color stair is composed of 6 color bars and 7 achromatic bars. The color sequence from left to middle on each color stair is m (magenta), c (cyan), y (yellow), b (blue), g (green), and r (red), and the achromatic bars from middle to right are k (black), 0.8k (80% gray), 0.6k (60% gray), 0.4k (40% gray), 0.2k (20% gray), 0.1k (10% gray), and w (white). The corresponding setting L*, a*, and b* values of the above test colors are shown in Table 2**.**

### 2.2. Paper-Based 3D Printing Parameters Settings

The 3D printing parameters consist of color conversion, slicing, and format conversion of 3D models. According to the color bar settings listed in Table 2, the color texture output was generated by Adobe Photoshop software CS6 (Adobe Systems, San Jose, CA, US)**,** and was pasted by Autodesk 3ds Max software to the surface of the model and saved as the “.obj” file format. Finally, the Mcor Orange conversion software (Version 2.0, Mcor-technologies, Dublin, Ireland) converted the L*, a*, and b* values into C, M, Y, K chromaticity values to create the “.mcor” file format that the Mcor ArkePro 3D printer can recognize.

### 2.3. Color Measurement, Imaging Acuqirtion, and Quantification

The sample chromaticity was measured by the exact spectrophotometer with M1 mode and a D50 light source to measure each color bar three times to obtain the average value. The original acquired images for image-based quality assessment were captured at a distance of 75.2 mm from the main view plane of each 3D test model with the D65 light source. The target color bars and white blocks were manually extracted and segmented into objects of the same size for further objective quantitative evaluation in the compiled MATLAB version R2016a program including the FSIMc (feature similarity index measure of color image) and iCID (improved color-image-difference) metrics. Subsequently, the composition of the featured information of the surface microscopic images at 100 magnification was analyzed to explain the changes in the objective metric described above.

### 2.4. Data Analysis

Based on our MATLAB program, the ΔE^*^_ab_, FSIMc, and iCID values were calculated from Sample 2 to Sample 10 compared with Sample 1, respectively. The specific calculation formulas are shown in the following Equations (1)–(3).

(1) $$\Delta E{*}_{\mathrm{ab}}\;=\;\sqrt{{\left(L^{*}{}_{x}-L^{*}{}_{y}\right)}^{2}+{\left(a^{*}{}_{x}-a^{*}{}_{y}\right)}^{2}+{\left(b^{*}{}_{x}-b^{*}{}_{y}\right)}^{2}}$$

where ΔE*_ab_ is the CIEDE 1976 color difference value; the L^*^_x_, a^*^_x_, b^*^_x_ are the chromaticity values of the test sample; and L^*^_y_, a^*^_y_, b^*^_y_ are the chromaticity values of the reference sample.

(2) $$FSIMc=\frac{\sum_{\Omega }S_{PC}\left(x\right)\cdot S_{G}\left(x\right)\cdot {\left[S_{I}\left(x\right)\cdot S_{Q}\left(x\right)\right]}^{\lambda }\cdot PC_{m}\left(x\right)}{\sum_{\Omega }PC_{m}\left(x\right)}$$

(3) $$iCID_{A\;}\left(S,T\right)=1-\frac{1}{\left|A\right|}\underset{i\in A}{\sum }\left[l_{L}\left(s_{i},t_{i}\right)\cdot c_{L}\left(s_{i},t_{i}\right)\cdot s_{L}\left(s_{i},t_{i}\right)\cdot l_{C}\left(s_{i},t_{i}\right)\cdot l_{H}\left(s_{i},t_{i}\right)\cdot c_{C}\left(s_{i},t_{i}\right)\cdot s_{C}\left(s_{i},t_{i}\right)\right]$$

For the meanings of the corresponding parameters and letter symbols in Equations (2) and (3), see References [31,32]. In our MATLAB program, we call the “FeatureSIM (imageRef, imageDis)” function and “iCID (Img1, Img2)” function to calculate the similarity and difference between the two images.

## 3. Results and Analysis

### 3.1. Effect of the Coloring Position on Paper-Based Model Surface on its Color Difference

**Figure 2:**
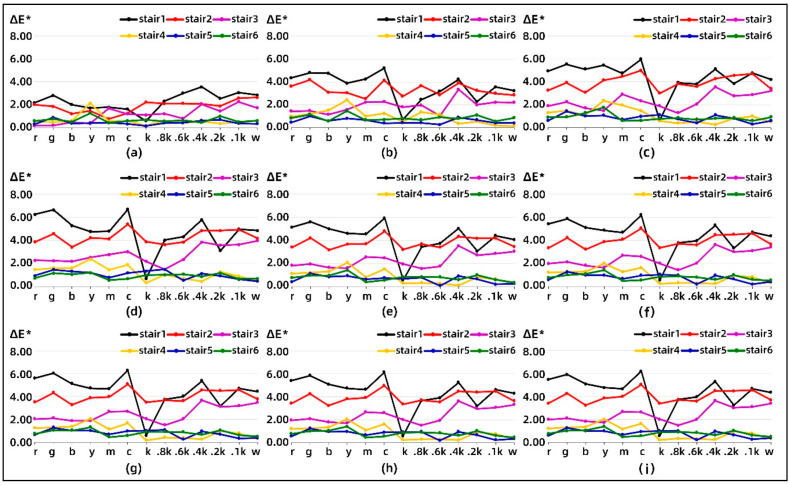
Comparison of the color difference between the multi-layer sample and Sample 1: (**a**) 2 layers, (**b**) 3 layers, (**c**) 4 layers, (**d**) 5 layers, (**e**) 6 layers, (**f**) 7 layers, (**g**) 8 layers, (**h**) 9 layers, and (**i**) 10 layers.

Figure 2 shows the color difference between the nine test samples and Sample 1 with only one printed layer on different color stairs. Except for Sample 2, the overall trend of the color difference distribution exhibited by the color bars on the other samples was relatively stable but also showed large fluctuations on individual color bars. Meanwhile, as the color stair is positioned higher, the relative color difference of its corresponding color bars behaves less and tends to stabilize on color stair 5 and stair 6. It is worth noting that the black bars on color stair 1 exhibit very little color difference for any change in the number of printed layers in each stair.

**Figure 3:**
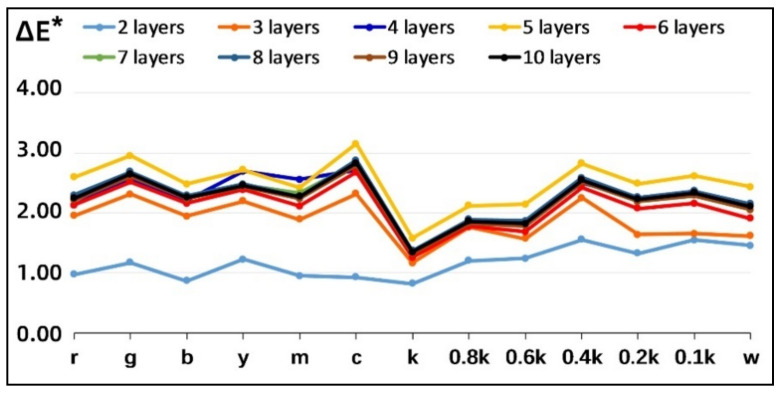
Average color difference of nine controlled combinations.

In Figure 3, it is possible to focus more clearly on the effect of the stair position on the mean value of the relative color difference evaluation. A significant correlation can be found between the average color difference and the number of printed layers per stair, which first increases and then decreases. Moreover, the average color difference remains slightly fluctuating overall after printing more than 6 layers per stair. The variation in the average color difference of the color bars is more stable than that of the non-colored bars at the same position of each stair.

Combined with the extreme value distribution of color difference for the selected colors listed in Table 3, it is obvious that most of the color stairs in Sample 2 have the smallest average value of the color bars on them, followed by Sample 6. Except for Sample 2, the minimum values of the average color difference of the colored stairs were mainly distributed in the white bars and 0.2k bars. Meanwhile, most of the color stairs in Sample 5 have the largest average value of the color bars on them, followed by Sample 4 and Sample 3. From a statistical point of view, this also somewhat validates the same trend between color difference and the number of printed layers for each stair.

### 3.2. Quantitative Analysis of the Image-Based Metrics of the Test Models

**Figure 4:**
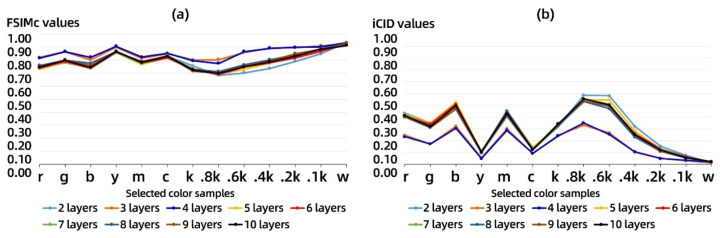
The average value of image-based metrics of test sample: (**a**) distribution of the feature similarity index measure of color image (FSIMc) values for nine controlled combinations and (**b**) distribution of the improved color-image-difference (iCID) values for nine controlled combinations.

The distribution of the average FSIMc values and iCID values for nine controlled combinations with increasing number of layers printed on the samples is shown in Figure 4. The FSIMc curve reflects the changing trend in the similarity degree of the details of the acquired images. The closer its value is to 1, the higher the similarity between two acquired images. The iCID curve reflects the changing trend in the visual difference degree of the acquired images, and the closer the value is to 0, the smaller the visual difference between two acquired images. 

As shown in Figure 4a, the FSIMc values of the 13 color bars show a consistent overall trend on all printed models but with slight fluctuations on individual color bars. For the achromatic bars, except for samples 3 and 4, the other samples show a linear trend with the FSIMc values of Sample 1. In Figure 4b, the overall trend of iCID values in all control combinations is similar to that of the FSIMc values but the trend of individual changes corresponding to iCID values fluctuates more. In addition, the iCID values of the achromatic bars change smoothly with respect to that of corresponding color bars, showing a parabolic trend and reaching a peak at the 0.8k color bar. By comparing the FSIMc values and iCID values of same color bars, it can be found that the color samples y and c fluctuate very much. At the same time, there is a negative correlation between the FSIMc values and iCID values of the grayscale color samples less than 0.8k in achromatic bars, and these correlation trends also exist in Samples 3 and 4. The average ΔE^*^_ab_ values, FSIMc values, and iCID values for each color stair against nine controlled combinations are demonstrated in detail in Table 4.

The numerical correlation between ΔE^*^_ab_ and FSIMc values and the relative correlation between ΔE^*^_ab_ and iCID values can be clearly compared for each controlled combination in Table 4. To this end, in Table 5, the quantitative correlation between the three objective metrics described above was further determined by averaging again the objective metrics for all test models containing different numbers of printed layers per color stair. This presented a more stable trend of linear correlation between the color difference and the image-based metrics, but the confidence of the linear fit with all color samples involved is not high.

From the above color difference correlation analysis in Section 3.1, since not all color samples show a consistent trend, it is necessary to further select color samples that are more consistent overall with the coloring position to quantify the trend in their image-based metrics. Herein, the color samples including r, g, b, m, and 0.4k were selected for linear fitting and established the linear equations between the overall average ΔE^*^_ab_ values, FSIMc values, and iCID values.

**Figure 5:**
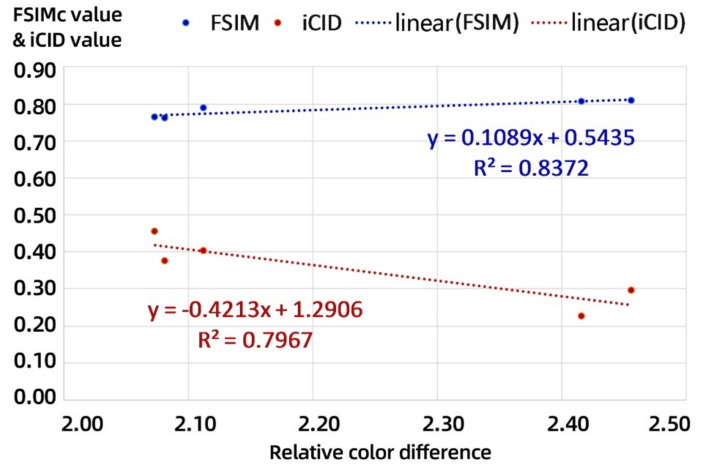
Linear fitting equations for specific color samples on all test models.

In Figure 5, the horizontal axis is the overall average ΔE^*^_ab_ values and the vertical axis is the corresponding FSIMc values and iCID values. The overall average ΔE^*^_ab_ values of the selected five color samples shows a high linear correlation with the overall average FSIMc values with a goodness-of-fit (equivalent to the coefficient of R square) of 0.8; its linear correlation with the overall average iCID values is relatively lower. However, the former is a positive linear correlation and the latter is a negative linear correlation. These feature provides an objective basis for the evaluation of full-color 3D printing surface coloring efficiency for image-based metrics using our 3D test charts, but their corresponding linear correlation equations in machine vision applications need to be calibrated according to the relevant coloring materials.

Therefore, the coloring quality evaluation of a full-color 3D-printed surface can be evaluated using image-based offline photography. This is because image-based metrics count the entire print surface, while the color difference measurement mostly requires local feature points. Obviously, based on the current 3D test chart and printing process, the linear fit of the color difference and image-based metrics is not yet better than 0.9, so the detailed characteristics of the microscopic surface coloring still need to be specifically analyzed.

### 3.3. Micrograph Analysis

**Figure 6:**
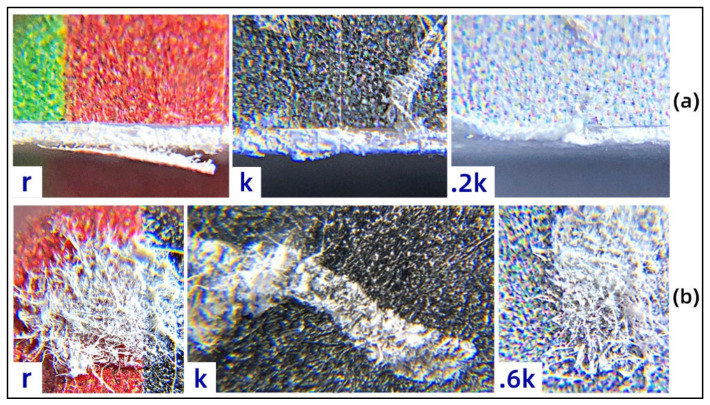
Surface microscopic defects of paper-based full-color 3D prints: (**a**) microscopic whitening defects; (**b**) microscopic fiber adhesion defects.

Due to the spatial network structure of the paper substrate consisting of fibers and the sticky fluctuation of the water-based adhesive, microscopic defects appear on the color surface of the actual 3D-printed parts. This can cause a low degree of linear correlation between the image-based metrics and the color difference on the full-color 3D-printed surface. For this reason, the typical microscopic color defect imaging of the test models is given in Figure 6. In Figure 6a, the microscopic whitening defects at the boundary of the colored layer are not easily detected in the main view but can be detected in the side view. This phenomenon occurs on both colored and gray samples and is related to border cutting accuracy and ink permeability. In Figure 6b, microscopic fiber adhesion defects also occurred in the surface microscopy of 13 basic color samples of the current 10 print models, such as the r, k, and 0.6k color samples. Such microscopic defects are mainly caused by unevenly sprayed adhesives. Another distinctive feature is that these microscopic defects occur mostly at the layer boundaries and at color mutations.

**Figure 7:**
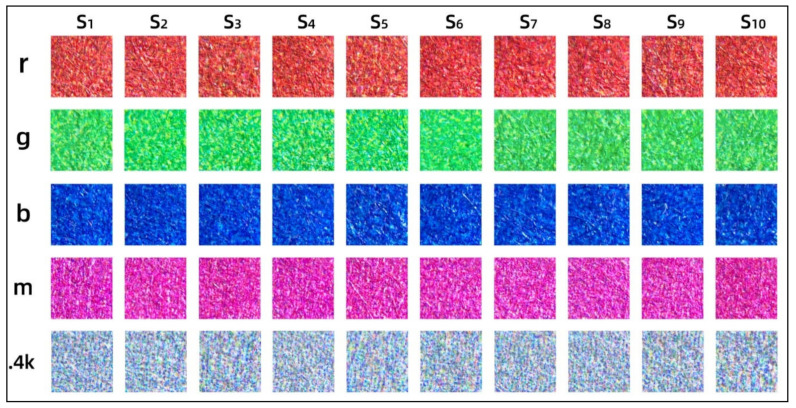
The microscopic images of the same surface sampling on each model.

In Figure 7, we further compared the microscopic imaging features of the surfaces of the five color samples used in the above correlation analysis. The microscopic images of different color samples of the same model can be found to be slightly different in human eye vision when the mixing state of the pigments can be seen from specific small dots. The most noticeable difference is the shape and area of the white areas in the image, which are caused by the different states of fiber accumulation. Since the pigment only accumulates in the hollow formed by the fibers and does not penetrate into the interior, the light reflected from the same sampled area varies, resulting in fluctuations in color difference and image-based metrics. This explains why the linear correlation between color difference and image-based metrics is not yet close to 1.

Analogously, the differences in the microscopic images of the same color samples compared to different models show not only white area changes but also perceptual differences in color area. For example, the perceptual differences with the r color bar of Sample 1 are Sample 2 and Sample 6; the perceptual differences with the g color bar of Sample 1 are Sample 6 and Sample 8; the perceptual differences with the b color bar of Sample 1 are Sample 6 and Sample 9; the perceptual differences with the m color bar of Sample 1 are Sample 5 and Sample 10; and the perceptual differences with the 0.4k color bar of Sample 1 are Sample 9 and Sample 10. These perceptual differences are mainly minor perceptible changes in lightness or saturation, which also lead to fluctuations in objective color differences associated with different print models. 

## 4. Discussion and Conclusions

In general, the colored surface of full-color paper-based 3D printing consists of a multi-fiber network structure and multi-colored pigment accumulation. However, small fluctuations in fiber length and distribution on the paper surface, pigment accumulation state, and inter-paper adhesion can also cause microscopic variation on the color surface. Micrograph analysis gives the overall physical differences but does not quantify individual surface properties. For the material community, colorimetric measurement and evaluation are easy to understand and operate. However, image-based quality evaluation involves computer graphics, image processing, and relative assessment algorithm development, which are challenges for scholars in materials chemistry and acceptable for scholars in computational materials. In addition, online measurement of the color 3D-printed parts is also an urgent need. Image-based acquisition and relative measurement analysis are available method references. Therefore, we correlated the color difference with the image-based metric to explore the color reproduction evaluation of paper-based color 3D printing. Moreover, we provided quantitative analysis for the evaluation of the surface coloring quality of 3D-printed parts from the material surface properties. 

In Table 6, we show the three surface properties of the overall white blocks on each printed model: whiteness, roughness, and glossiness. The first thing that can be noticed is that the average whiteness of the individual models fluctuates between 92–93 ISO whiteness, even though the white blocks are printed by paper that is cut from the same roll. Since the image acquisition of the main view plane of the printed model contains six white blocks, the acquired image-based metrics are also influenced by the whiteness of the white blocks. The average whiteness of the white blocks of Samples 3 and 4 in this table is very similar to that of Sample 1, which in turn explains to some extent the stable trend in the corresponding FSIMc values and iCID values in Figure 4.

Compared with the Sample 1, the roughness trend of white works is not as regular for all test models, while the overall deviation range is within 0.03. The samples with roughness differences from Sample 1 are mainly Sample 5, Sample 6, Sample 9, and Sample 10, which are similar to the perceptual difference variation of microscopic images. The greater surface roughness of the white block indicates that the surface roughness of the current paper before coloring is greater and that the pigment filling characteristics are altered, but there are no specific research papers describing this mechanism. From the current distribution of gloss values, the glossiness of the white block surface appears to be inversely proportional to the number of printed layers. However, combined with the fractional accuracy of the gloss meter, the glossiness of white blocks made from stacks of the same paper is subject to small fluctuations, but there is no strict linear correlation. These fluctuations may be due to microscopic surface fiber adhesion defects.

For current printing paper substrates, the roughness and gloss are low and the whiteness is high, which ensures the accuracy of the chromaticity measurement and thus provides an objective reference for the quantitative evaluation of the relative color difference of different printing models. In paper-based full-color 3D printing, the print surface whiteness, roughness, and gloss depend on its raw material grade, which can be considered as constant properties before printing. However, the bonding quality and postprocessing involved after printing can make the printed surface properties change randomly, as verified in the microscopic image analysis above.

Overall, assessing the surface color quality based on color difference or image-based metrics using our designed 3D test charts can provide quantitative prediction of the surface coloring efficiency for color 3D printing devices or coloring substrates. Its prediction accuracy depends on the degree of linear correlation between the two types of evaluation metrics, although the current goodness-of-fit is around 0.8. From the qualitative analysis of microscopic images, the current linear goodness of fit of both can also be improved by the elimination of microscopic defects. However, quantitative assessment of the microscopic properties of the stair surface at different locations of the test model for predicting the coloring efficiency of the pretreated paper-based surface needs further study. In addition, the correlation between color difference and image-based metric shows a good linear trend in 3D color test charts with height variations with five test points for specific flat surfaces but remains to be verified and modeled in 3D color test charts corresponding to the top curved surfaces.

## Figures and Tables

**Table 1:** Printing layers and thickness of each paper-based 3D model.

Unit of Thickness: mm		White Blocks						Color Bars					
Sample ID	Printing Stairs	W_s1_	W_s2_	W_s3_	W_s4_	W_s5_	W_s6_	C_s1_	C_s2_	C_s3_	C_s4_	C_s5_	C_s6_
1 (S_1_)	C×1+W×1	0.1	0.3	0.5	0.7	0.9	1.1	0.2	0.4	0.6	0.8	1.0	1.2
2 (S_2_)	C×1+W×3	0.3	0.7	1.1	1.5	1.9	2.3	0.4	0.8	1.2	1.6	2.0	2.4
3 (S_3_)	C×1+W×5	0.5	1.1	1.7	2.3	2.9	3.5	0.6	1.2	1.8	2.4	3.0	3.6
4 (S_4_)	C×1+W×7	0.7	1.5	2.3	3.1	3.9	4.7	0.8	1.6	2.4	3.2	4.0	4.8
5 (S_5_)	C×1+W×9	0.9	1.9	2.9	3.9	4.9	5.9	1.0	2.0	3.0	4.0	5.0	6.0
6 (S_6_)	C×1+W×11	1.1	2.3	3.5	4.7	5.9	7.1	1.2	2.4	3.6	4.8	6.0	7.2
7 (S_7_)	C×1+W×13	1.3	2.7	4.1	5.5	6.9	8.3	1.4	2.8	4.2	5.6	7.0	8.4
8 (S_8_)	C×1+W×15	1.5	3.1	4.7	6.3	7.9	9.5	1.6	3.2	4.8	6.4	8.0	9.6
9 (S_9_)	C×1+W×17	1.7	3.5	5.3	7.1	8.9	10.7	1.8	3.6	5.4	7.2	9.0	10.8
10 (S_10_)	C×1+W×19	1.9	3.9	5.9	7.9	9.9	11.9	2.0	4.0	6.0	8.0	10.0	12.0

**Table 2:** The original test colors in the experiment and corresponding chromaticity values.

Color	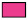	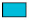	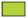	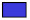	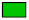	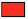	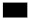	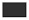	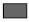	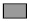	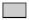	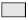	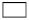
	m	c	y	b	g	r	k	0.8k	0.6k	0.4k	0.2k	0.1k	w
L*	50	70	80	35	70	50	0	21	43	63	82	91	100
a*	60	−30	−20	40	−60	65	0	0	0	0	0	0	0
b*	−10	−25	60	−80	60	50	0	0	0	0	0	0	0

**Table 3:** Distribution of extreme values of color difference for nine controlled combinations.

	Minimum Distribution						Maximum Distribution					
	Stair1	Stair2	Stair3	Stair4	Stair5	Stair6	Stair1	Stair2	Stair3	Stair4	Stair5	Stair6
r	S_2_	S_2_	S_2_	S_2_	S_2_	S_2_	S_5_	S_5_	S_5_	S_5_	S_5_	S_4_
g	S_2_	S_2_	S_2_	S_2_	S_2_	S_2_	S_5_	S_5_	S_5_	S_5_	S_5_	S_5_
b	S_2_	S_2_	S_2_	S_2_	S_2_	S_2_	S_5_	S_5_	S_5_	S_5_	S_5_	S_4_
y	S_2_	S_2_	S_2_	S_7_	S_2_	S_5_	S_4_	S_5_	S_5_	S_3_	S_5_	S_4_
m	S_2_	S_2_	S_2_	S_2_	S_2_	S_6_	S_5_	S_4_	S_4_	S_4_	S_5_	S_3_
c	S_2_	S_2_	S_2_	S_2_	S_2_	S_6_	S_5_	S_5_	S_5_	S_5_	S_5_	S_3_
k	S_6_	S_2_	S_2_	S_2_	S_2_	S_2_	S_3_	S_5_	S_5_	S_4_	S_5_	S_5_
0.8k	S_2_	S_2_	S_2_	S_9_	S_2_	S_2_	S_5_	S_4_	S_3_	S_3_	S_5_	S_5_
0.6k	S_2_	S_2_	S_2_	S_6_	S_6_	S_2_	S_5_	S_5_	S_5_	S_3_	S_5_	S_5_
0.4k	S_2_	S_2_	S_2_	S_6_	S_2_	S_2_	S_5_	S_5_	S_5_	S_2_	S_5_	S_5_
0.2k	S_3_	S_2_	S_2_	S_2_	S_7_	S_4_	S_4_	S_5_	S_5_	S_5_	S_5_	S_5_
0.1k	S_2_	S_2_	S_2_	S_3_	S_6_	S_2_	S_5_	S_5_	S_5_	S_3_	S_5_	S_5_
w	S_2_	S_2_	S_2_	S_3_	S_6_	S_6_	S_5_	S_5_	S_5_	S_2_	S_4_	S_4_

**Table 4:** Averaged ΔE^*^_ab_, FSIMc, and iCID values of nine controlled combinations.

Layers	Metrics	r	g	b	y	m	c	k	0.8k	0.6k	0.4k	0.2k	0.1k	w
2L	ΔE^*^_ab_	0.97	1.16	0.86	1.22	0.95	0.92	0.82	1.20	1.24	1.55	1.32	1.54	1.45
	FSIMc	0.74	0.78	0.74	0.86	0.79	0.83	0.76	0.68	0.70	0.74	0.79	0.85	0.93
	iCID	0.43	0.35	0.51	0.10	0.41	0.12	0.32	0.59	0.58	0.32	0.15	0.08	0.02
3L	ΔE^*^_ab_	1.95	2.31	1.94	2.20	1.89	2.32	1.16	1.76	1.57	2.25	1.64	1.65	1.61
	FSIMc	0.82	0.87	0.80	0.90	0.82	0.85	0.80	0.80	0.86	0.89	0.90	0.91	0.93
	iCID	0.25	0.17	0.32	0.05	0.30	0.09	0.25	0.33	0.27	0.10	0.05	0.03	0.01
4L	ΔE^*^_ab_	2.13	2.58	2.20	2.70	2.55	2.71	1.32	1.83	1.83	2.50	2.26	2.36	2.12
	FSIMc	0.82	0.87	0.82	0.91	0.82	0.85	0.80	0.77	0.87	0.89	0.90	0.90	0.93
	iCID	0.23	0.17	0.31	0.05	0.29	0.09	0.24	0.35	0.25	0.11	0.05	0.03	0.02
5L	ΔE^*^_ab_	2.59	2.96	2.48	2.72	2.42	3.15	1.57	2.12	2.14	2.83	2.48	2.62	2.44
	FSIMc	0.73	0.79	0.74	0.86	0.77	0.82	0.73	0.69	0.73	0.78	0.83	0.89	0.92
	iCID	0.42	0.35	0.53	0.11	0.45	0.14	0.32	0.55	0.55	0.28	0.13	0.06	0.03
6L	ΔE^*^_ab_	2.12	2.52	2.16	2.39	2.11	2.68	1.26	1.78	1.68	2.43	2.07	2.15	1.91
	FSIMc	0.74	0.79	0.74	0.86	0.78	0.82	0.73	0.69	0.75	0.78	0.82	0.87	0.93
	iCID	0.41	0.34	0.51	0.10	0.44	0.13	0.33	0.56	0.50	0.26	0.13	0.06	0.02
7L	ΔE^*^_ab_	2.22	2.64	2.25	2.47	2.32	2.83	1.35	1.86	1.82	2.54	2.24	2.34	2.12
	FSIMc	0.75	0.80	0.76	0.86	0.78	0.84	0.73	0.70	0.75	0.80	0.84	0.88	0.93
	iCID	0.42	0.32	0.49	0.11	0.45	0.13	0.32	0.56	0.51	0.24	0.13	0.06	0.02
8L	ΔE^*^_ab_	2.30	2.69	2.29	2.48	2.27	2.88	1.37	1.89	1.86	2.59	2.26	2.37	2.15
	FSIMc	0.76	0.80	0.78	0.87	0.78	0.83	0.73	0.71	0.76	0.80	0.84	0.89	0.93
	iCID	0.41	0.33	0.47	0.10	0.45	0.13	0.33	0.53	0.47	0.23	0.11	0.05	0.02
9L	ΔE^*^_ab_	2.21	2.61	2.23	2.44	2.23	2.79	1.32	1.83	1.78	2.52	2.19	2.29	2.05
	FSIMc	0.75	0.80	0.76	0.86	0.79	0.83	0.72	0.70	0.76	0.79	0.85	0.88	0.93
	iCID	0.40	0.31	0.47	0.10	0.41	0.13	0.35	0.54	0.49	0.25	0.11	0.06	0.02
10L	ΔE^*^_ab_	2.24	2.64	2.26	2.46	2.27	2.83	1.34	1.86	1.82	2.55	2.23	2.33	2.10
	FSIMc	0.74	0.80	0.74	0.87	0.78	0.83	0.72	0.70	0.75	0.79	0.83	0.88	0.91
	iCID	0.41	0.32	0.50	0.10	0.43	0.12	0.34	0.56	0.50	0.25	0.12	0.06	0.02

**Table 5:** Comparison of overall relative ΔE^*^_ab_, FSIMc, and iCID values of all test samples.

	r	g	b	y	m	c	k	0.8k	0.6k	0.4k	0.2k	0.1k	w
ΔE^*^_ab_	2.08	2.46	2.07	2.34	2.11	2.57	1.28	1.79	1.75	2.42	2.08	2.18	1.99
FSIMc	0.76	0.81	0.77	0.87	0.79	0.83	0.75	0.72	0.77	0.81	0.84	0.88	0.93
iCID	0.38	0.30	0.45	0.09	0.40	0.12	0.31	0.51	0.46	0.23	0.11	0.05	0.02

**Table 6:** Whiteness, roughness, and glossiness of white blocks for all test models.

Paper	S_1_	S_2_	S_3_	S_4_	S_5_	S_6_	S_7_	S_8_	S_9_	S_10_
Whiteness	92.401	92.732	92.572	92.556	92.720	92.624	92.717	92.709	92.686	92.721
Roughness	3.150	3.152	3.143	3.144	3.166	3.157	3.145	3.146	3.162	3.163
Glossiness	4.2	4.1	4.0	4.1	4.0	4.2	4.0	3.8	3.7	3.8

## Data Availability

Data is contained within the article.
